# Trajectory of Obesity and the Impact of Eating Behaviors on Obesity in Preschool Children: A Nationwide Population-Based Cohort Study in Korea

**DOI:** 10.3390/children11111297

**Published:** 2024-10-27

**Authors:** Kyeong Hun Lee, Baek Seung Kim, Gitae Seo, Hyeon-Jong Yang, Kyunghoon Kim, Eun-Ae Yang

**Affiliations:** 1 Department of Pediatrics, Seoul St. Mary’s Hospital, College of Medicine, The Catholic University of Korea, Seoul 06591, Republic of Korea; lkhoon88@cmcnu.kr; 2 Department of Pediatrics, Seoul National University Bundang Hospital, Seoul 13620, Republic of Korea; kbs778@naver.com; 3 SCH Biomedical Informatics Research Unit, Soonchunhyang University, Seoul Hospital, Seoul 31538, Republic of Korea; gtseo@schmc.ac.kr (G.S.); pedyang@schmc.ac.kr (H.-J.Y.); 4 Department of Pediatrics, Soonchunhyang University Seoul Hospital, Soonchunhyang University College of Medicine, Seoul 31538, Republic of Korea; 5 Department of Pediatrics, Seoul National University College of Medicine, Seoul 03080, Republic of Korea; 6 Department of Pediatrics, Daejeon St. Mary’s Hospital, College of Medicine, The Catholic University of Korea, Daejeon 34943, Republic of Korea; 7 Department of Pediatrics, Chungnam National University Hospital, Chungnam National University School of Medicine, Daejeon 35015, Republic of Korea

**Keywords:** obesity, children, eating behaviors, risk factor, trend

## Abstract

**Background**: Early childhood obesity is highly associated with obesity in adolescents and adults. However, studies on specific eating behaviors that have a decisive effect on obesity in early childhood (aged 3–5 years) are scarce. We hypothesized that critical risk factors associated with eating behaviors leading to obesity at ages of 3–5 years may be different. To confirm this hypothesis, we conducted a study on the risk factors of obesity related to eating behaviors in preschool children. **Methods**: Using the National Health Screening Program for Infants and Children (NHSPIC) in Korea’s general population cohort, we found the obesity trends in 3–5-year-olds. The risk factors of obesity in 3–5-year-olds were analyzed through logistic regression. **Results**: Among children with obesity at 3 years old, but without obesity at 4 years old, only 10.1% (107/1063) transitioned to obesity at 5 years old, whereas among children with obesity at 3–4 years old, 62.7% (398/635) transitioned to obesity at 5 years old. The risk factors for obesity at 3 years old were inclusion of meat in complementary food, prolonged breastfeeding, and consumption of sweetened beverages; at ages 4–5 years, the risk factors were the inclusion of meat in complementary food and consumption of sweetened beverages. **Conclusions**: Obesity at 4 years is more likely to lead to obesity in the following year than obesity at 3 years. The dietary factor with the greatest impact on obesity in children aged 3–5 years has been found to be consumption of sweetened beverages.

## 1. Introduction

Childhood obesity is an important global health issue, and its prevalence is increasing [1]. Childhood obesity is associated with several complications, including metabolic and cardiovascular disorders [2]. Additionally, early childhood obesity is highly associated with obesity in adolescents and adults [3]. Among adolescents with obesity, the greatest acceleration in annual body mass index (BMI) increments occurred between the ages of 2 and 6 years; a higher acceleration in preschool annual BMI increments is associated with a risk of overweight or obesity in adolescence [3]. A representative British cohort study that measured the BMI at the ages of 3, 5, 7, and 11 years reported that the obesity group had a clear trend towards obesity from the age of 3 years [4]. For this reason, early intervention within the family to prevent obesity from childhood is very important for lifelong health.

Obesity is a result of the interaction between positive energy intake, decreased physical activity, intrauterine growth, socioeconomic status, and genetic factors [5,6]. The dietary intake habits of young children may play an important role in increasing the prevalence of childhood obesity globally [7]. In particular, in Korea, economic prosperity and westernized eating habits have made eating habits a very important cause of childhood obesity. Eating behavior begins to solidify at the age of 3 years and is almost completely formed by the time of entering school [8]. Despite this, most parents are not making efforts to improve eating habits in order to prevent obesity in young children. In addition, although there are many studies on the factors causing obesity, large-scale studies on eating habits that have a decisive influence on obesity in young children are limited [9,10].

Accordingly, this study was conducted as part of an evidence collection effort targeting a large cohort group of toddlers and preschool children to identify the eating habits that have the greatest influence on obesity of toddlers and preschool children. The purpose of this study was to identify the eating habits that should be considered as a priority to prevent obesity in infants to preschool children, and to help educate caregivers to prevent childhood obesity, as the eating habits of young children are ultimately determined by parents and families.

## 2. Methods

This cohort study used the Korean National Health Insurance Service (NHIS) medical database and the National Health Screening Program for Infants and Children (NHSPIC) in Korea. A 5% sample for each birth year was randomly extracted nationwide from all examinees who received the 1st and 2nd NHSPIC for infants and young children born between 2008 and 2012. An NHSPIC database was established for infants and young children using a cohort method, from 2008 to 2015, by combining it with the NHIS. This study is a large-scale domestic birth cohort study based on NHSPIC data, the most extensive database on health care for children in Korea. The NHSPIC consisted of 7 surveys administered at ages of 4–72 months (1st: 4–6 months; 2nd: 9–12 months; 3rd: 18–24 months; 4th: 30–36 months; 5th: 42–48 months; 6th: 54–60 months; and 7th: 66–71 months), and included information on physical examination, developmental screening, general and oral health questionnaires, and parenting guidance [11].

The 7 surveys of NHSPIC provide nutritional questionnaire information, anthropometric indices (weight, height, body mass index, and head circumference), birth history, lactation, developmental status, socio-demographic information (sex, age, residence, disability, death, and medical security), and medical claim data (medical institution use, medical expenses, and diagnoses) [12].

BMI [weight (kg)/height^2^ (m^2^)] was calculated using body weight (kg) and height (m) in NSHPIC data. BMI percentile by sex and age was evaluated based on the 2017 Korean National Growth Charts published by the Korean Centers for Disease Control and Prevention and the Korean Pediatric Society [13]. If the BMI is above the 95th percentile, it is classified as obese. If it is above the 90th percentile but below the 95th percentile, it is classified as overweight. If it is below the 90th percentile, it is classified as normal weight. Independent variables were selected from the NHSPIC by considering the main factors that may affect childhood obesity. Variable selection was based on previous studies and literature reviews [14,15,16,17]. The variables consisted of questionnaire answers from the respective NHSPIC. ‘Prematurity’ was defined as the case where “yes” was indicated on the item “Was the child born premature?”. ‘Prolonged breastfeeding’ referred to breastfeeding for 9 months or more. ‘Late complementary food’ refers to cases where complementary food was introduced after 7 months of age, and ‘Complementary food (≥3/day) refers to the intake of complementary food at least three times a day during 9–12 months. ‘Other foods’ refers to ‘sunsik’, which is a cereal-based ready-to-drink Korean beverage, yogurt, or honey consumed during the first 9–12 months of life. ‘Sugar-sweetened beverages’, such as fruit juice or other artificial sugar-containing beverages, were defined as the consumption of more than 200 mL in 18–24 months (3rd), 30–36 months (4th), 42–48 months (5th) of life. ‘Meal (≥4/day)’ was defined as four or more meals at 30–36 months (4th) and 42–48 months (5th) of age.

This study included infants who underwent the NHSPIC 1st stage survey from 2008 to 2010. The data were excluded if a disability diagnosis code according to international classification of diseases 10th revision was received or if any of the 1st to 7th stage NHSPIC had not been performed. Statistical analyses were conducted using the SAS Enterprise Guide (version 7.1) and R Studio (version 1.0.136) and were based on a 2-tailed *p*-value.

A logistic regression model was utilized to analyze the risk factors for obesity, with obesity being treated as a discrete variable dependent on normal (0) versus overweight/obesity (1), and normal/overweight (0) versus obesity (1). Multicollinearity was assessed using the variance Inflation Factor (VIF), which was found to be less than five, indicating no risk of multicollinearity. Relative risk (RR) and the confidence interval (CI) was recorded. Additionally, the results of the homogeneity test using the chi-squared test for each variable in the population by age were recorded.

## 3. Results

A total of 122,650 subjects were extracted from the NSHPIC. Of these, a total of 16,866 were analyzed in this study, excluding those who received a disability diagnostic code (*n* = 32) or had any missing stage data of NSHPIC (*n* = 105,752). The distributions of normal weight, overweight, and obesity were 13,184 (78.2%), 1984 (11.8%), and 1698 (10.1%) at 3 years of age; 14,179 (84.1%), 1638 (9.7%), and 1049 (6.2%) at 4 years of age; and 14,632 (86.8%), 1262 (7.5%), and 972 (5.8%) at 5 years of age (Supplementary Table S1).

**Figure 1:**
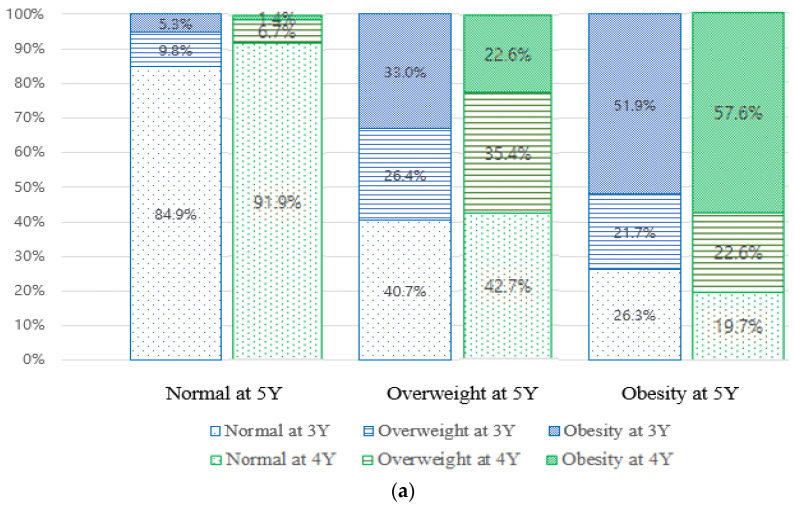

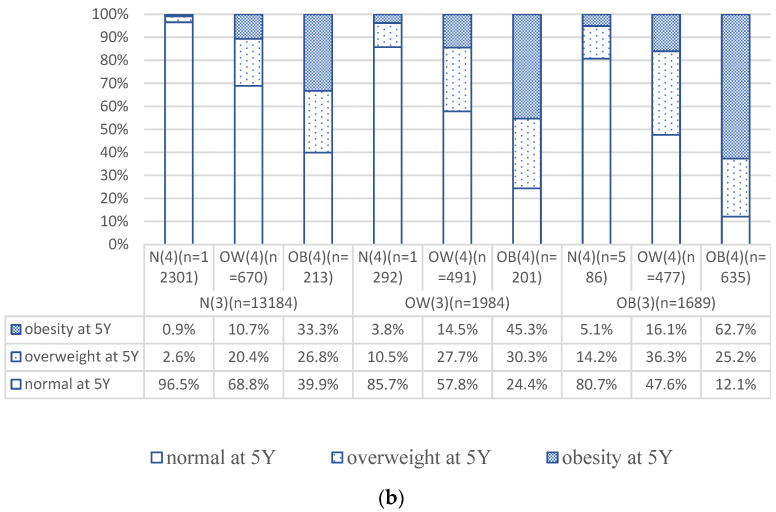
Trends in obesity at age of 3–5 years. (**a**) This figure shows trends in normal weight, overweight, and obesity at ages 3–5. (**b**) The group with obesity at both ages 3 and 4 was the most likely to have obesity at age 5 (62.7%), followed by the group with overweight at age 3 and obesity at age 4 (45.3%), and the group with normal weight at age 3 and obesity at age 4 (33.3%). Even if obesity was present at 3 years old, in the group with normal weight or overweight at age 4, 5.1% and 16.1% were confirmed to have obesity at age 5, respectively.

The proportion of female residents was 48.9% (8238/16,866), the proportion of residents living in cities was 42.1% (7085/16,818), and the proportion of low-income residents was 35.7% (6017/16,866). At 3 and 4 years of age, there was no difference in the obesity with respect to sex; however, at 5 years of age, girls appeared to have less obesity. Children of 4 and 5 years of age were more likely to be with obesity when living in the city. The low-household-income group had higher rates of non-obesity across all age groups. In the case of high birth weight (≥4 kg), the proportion of obesity was significantly higher at all ages. If there was a visual abnormality on the NSHPIC performed at the closest time point, the obesity ratio increased significantly across all age groups. The ratio of obesity at 3 and 4 years of age was significantly increased in the presence of auditory abnormalities on the fourth NSHPIC at 30–36 months and the fifth NSHPIC at 42–48 months, respectively (Table 1). Of 13,184 children of normal weight aged 3 years, 93.3% (12,301/13,184) of children were of normal weight and 5.1% (670/13,184) of children were overweight at 4 years of age. Of the 1984 children who were overweight at 3 years of age, 65.1% (1292/1984) of children were of normal weight, 24.7% (491/1984) of children were overweight, and only 10.1% (201/1984) had obesity at 4 years of age. However, of the 1698 children with obesity at 3 years of age, 34.5% (586/1698) of children became normal weight, 28.1% (477/1698) of children became overweight, and 37.4% (635/1698) of children became obese at 4 years of age. The proportion that transitioned, from 4 to 5 years of age, to normal weight, overweight, and obesity was as follows: normal weight at 4 years (*n* = 14,179) 94.9% were of normal weight, 3.8% had overweight, and 1.4% had obesity at 5 years of age. Among those who had overweight at 4 years of age (*n* = 1638), 59.3% were of normal weight, 29.2% had overweight, and 13.4% had obesity at 5 years of age. Among those who had obesity at 4 years of age (*n* = 1049), 20.1% were of normal weight, 26.5% of children had overweight, and 53.4% of children had obesity at 5 years of age. Among children who had obesity at the age of 3 years, but had normal weight or overweight at 4 years (62.6%, 1063/1698), only 10.1% transitioned to having obesity at 5 years of age, whereas among children who had obesity at 3 years and at 4 years (37.4%, 635/1698), 62.7% transitioned to be with obesity at 5 years of age (Figure 1).

In the figure, N represents normal weight, OW represents overweight, and OB represents obesity. And the number in parentheses indicates the age. Y; years-old

The risk factors for obesity at 3 years of age were the inclusion of meat in complementary food at 9–12 months, breastfeeding over 9 months, and consumption of sugar-sweetened beverages at 30–36 months (Table 2); the risk factors of obesity at 4 years of age were the inclusion of meat in complementary food at 9–12 months and consumption of sugar-sweetened beverages at 42–48 months (Table 3). The risk factors for obesity at 5 years of age were the inclusion of meat in complementary food at 9–12 months and consumption of sugar-sweetened beverages at 42–48 months (Table 4). Low birth weight, premature birth, and inclusion of grains in complementary food at 9–12 months were associated with reduced obesity at the age of 3 years (Table 2); low birth weight, intake of complementary foods (≥3/d) at 9–12 months and inclusion of grains in complementary food at 9–12 months were associated with reduced obesity at 4 years of age (Table 3); low birth weight and intake of complementary food (≥3/d) at 9–12 months were associated with reduced obesity at 5 years of age (Table 4). An analysis of overweight and obesity is presented in Supplementary Table S2. At all ages, low birth weight and intake of sweetened beverages had the same significant effects on overweight and obesity status. Notably, at 3 and 5 years of age, a frequency of meals of at least four times a day was identified as the strongest risk factor in the overweight and obesity groups.

## 4. Discussion

In this study, the prevalence of obesity was 10.1% in 3-year-olds, 6.2% in 4-year-olds, and 5.8% in 5-year-olds from 2008 to 2015. This is similar to the results of previous domestic studies that reported that the overall obesity prevalence increased from 5.8% in 1997 to 9.7% in 2005 among 183,159 children and adolescents aged 2 to 18 years, but obesity prevalence in children aged 2 to 6 was 5.1% in 1997, and 6.3% in 2005 in 2–6-year-olds [18]. In a domestic study that investigated the obesity rate in a small regional population of children aged 3 to 5 years old, Park et al. reported the obesity rate as 13.5% in 2007, and Ra et al. reported the obesity rate as 8.1% in 2017 [19,20]. In terms of global prevalence, the prevalence of obesity in children under 5 years of age was 7.8–8.2% in the United States from 2011 to 2012 and in Canada from 2000 to 2003, showing similar results regardless of time and region [21,22]. Unlike previous studies, this study was a cohort study rather than a cross-sectional study, and the prevalence of obesity was highest at 10.1% at age 3, and decreased to approximately 6% at ages 4 and 5, showing a similar prevalence rate. In addition, our results showed that obesity at 4 years of age can be much more closely linked to obesity at 5 years of age than obesity at 3 years of age. It can be hypothesized that obesity at ages 4 and 5 years may have a greater effect on the obesity rate in adolescents than at the age of 3 years. Therefore, the modulation of factors more influential on obesity at the age of 4 or 5 years, compared to at the age of 3 years. may be more useful in controlling obesity after adolescence, and strategies to prevent obesity can be established.

Among the factors contributing to the very early period, the rate of weight gain during the first year of life appears to be most strongly associated with childhood obesity [5]. Several reports demonstrate that birth weight plays a role in childhood obesity and overweight [9,23]. High birth weights (≥4.0 kg) were significantly higher in the obesity group than in the non-obesity group in all groups of 3–5-year-old children in our study. A high birth weight is the result of fetal hyperglycemia and hyperinsulinemia during pregnancy due to maternal overnutrition. These effects have been linked to the transition to obesity in adulthood, which can lead to type 2 diabetes and metabolic disease later in life [24,25]. Breastfeeding and dietary habits during early life have been known to influence the rates of obesity in childhood and adolescence [5,9]. Not only does breast-feeding itself reduce the risk of childhood obesity, but changing the duration of breastfeeding has also been shown to be protective against the prevalence of obesity [14]. Notably, our results showed that prolonged breastfeeding beyond 9 months had no effect on obesity at 4–5 years of age; however, it was identified as a risk factor for obesity at 3 years of age. Meat inclusion in complementary food was a risk factor for obesity in children aged 3–5 years; however, grain inclusion in complementary food protected against obesity in children aged 3–4 years. According to the European Society for Pediatric Gastroenterology Hepatology and Nutrition position paper, a high protein intake during weaning tends to lead to obesity and overweight later in life [15]. In contrast, the intake of grains in complementary food was identified as a protective factor against obesity in children aged 3 to 4 years. The Lancet committee also recommended a diet consisting of a low quantity of red meat and sugar and consisting largely of whole grains [6]. Additionally, grain consumption in infancy is known to have beneficial effects on health and development during infancy and early childhood [26].

Aggressive promotion of breast milk substitutes and increased marketing of high energy density and low-nutrient foods and beverages have contributed significantly to the global prevalence of childhood overweight and obesity [27,28]. Particularly, sugar intake is closely related to obesity in children as well as adults, and has been reported as a major cause of cardiovascular and metabolic diseases [29,30]. Sugar-sweetened beverages have also been reported as a major risk factor for obesity in preschool children [31,32]. In this study, the diet habit that commonly acts as the greatest risk factor for obesity in children aged 3, 4, and 5 was evaluated as regular sugar-sweetened beverage intake exceeding 200 mL per day. It is noteworthy that among various eating habits, sugar-sweetened beverage intake was reported to be the strongest dietary factor for obesity in all ages, 3, 4, and 5. Early childhood dietary characteristics are more highly influenced by caregivers, childcare facilities and environments, and social perceptions and culture, than by individual preferences [33,34]. However, addressing childhood obesity based on individualized education is not efficient, and most studies have failed [35,36]. Therefore, creating environments for healthy eating behavior will be key to addressing childhood obesity [37]. For this, there will be a need for active government involvement in ensuring that the recommended healthy nutritional diets, such as those including low sugar and high-in-grain foods, are consumed, and a clear healthy growth strategy will be required [6,28].

A key feature of our study is the use of a large nationwide cohort follow-up for a period of 4–71 months. In particular, our study showed how obesity changes with age in children, analyzing the factors associated with eating habits. However, there are some limitations. First, there are no data on whether this translates into obesity transitions in adolescence and adulthood; second is the lack of consideration of energy consumption, such as physical activity. However, the most of children are enrolled in daycare centers or kindergartens between 3 and 5 years of age and have similar energy expenditures in Korea; therefore, the dietary habits related to energy intake are thought to have a greater impact on obesity during early childhood. Third, the association of genetic factors in the development of obesity has been reported and the genetic association with obesity has been shown to be more pronounced with increased consumption of sugar-sweetened beverages [38,39,40,41]. However, genetic issues, such as parental obesity, were not considered in this study. Finally, since 5% of infants were selected as a sample, it may not represent the entire population. Additionally, infants from certain socioeconomic backgrounds may be under-represented or over-represented. To address this, an increase in the sample size and the use of proportional sampling from diverse backgrounds will be needed.

## 5. Conclusions

Our study showed changes in obesity at the ages of 3–5 years, with a more prevalent transition from obesity at 4 years to obesity in the following year than obesity at 3 years of age. The biggest contributing factor to obesity in children aged 3–5 years is the consumption of sweetened beverages. Additionally, control of current dietary habits and attention to dietary habits play important roles in protecting against childhood obesity.

## Figures and Tables

**Table 1:** Demographic characteristics of study population.

Characteristics	Age 3 y (*n*)				Age 4 y (*n*)			Age 5 y (*n*)	
	Non-Obesity	Obesity	*p* Value	Non-Obesity	Obesity	*p* Value	Non-Obesity	Obesity	*p* Value
	(15,168)	(1698)		(15,817)	(1049)		(15,894)	(972)	
**Birth weight**									
**HBW ^a^, %**	2.6	6.7	**<0.001**	2.7	7.4	**<0.001**	2.8	6.5	**<0.001**
**NBW, %**	76.3	73.4	**0.007**	76.2	73.5	0.05	76.1	74.7	0.327
**LBW ^a^, %**	21.1	19.9	0.283	21.1	19.1	0.133	21.1	18.8	0.103
**Sex**									
**Female, %**	48.6	51.1	0.054	48.7	48.6	0.879	49.1	45	**0.012**
**Residence**									
**City ^b^, %**	42.3	40.5	0.151	41.8	46.7	**0.002**	41.9	46.6	**0.04**
**Household income**									
**Low ^c^, %**	36.2	31.2	**<0.001**	36.1	29.6	**<0.001**	35.9	31.1	**0.02**
**Visual abnormality ^d^, %**									
**4th**	1.5	2.3	**0.017**	1.6	1.3	0.469	1.6	1.9	0.532
**5th**	1.8	2	0.398	1.7	2.9	**0.007**	1.7	2.8	**0.018**
**Auditory abnormality ^d^, %**									
**4th**	1.5	2.5	**0.005**	1.6	1.9	0.536	1.6	2.1	0.336
**5th**	1.8	2.1	0.454	1.7	2.9	**0.011**	1.7	2.8	**0.026**
**Developmental delay ^d^, %**									
**4th**	3.5	4.4	0.054	3.6	4.1	0.363	3.5	4.7	**0.049**
**5th**	2.3	2.5	0.608	2.3	3.2	0.05	2.3	3.2	0.076

Abbreviations: HBW, high birth weight; NBW, normal birth weight; LBW, low birth weight; 4th, questionnaire was conducted at 30–36 months; 5th, questionnaire was conducted at 42–48 months. Bold style indicates a *p* value < 0.05. This means it is statistically significant.

**Table 2:** Analysis by multivariable logistic regression on risk factors of eating behaviors, normal + overweight vs. obesity (Age of 3 years).

Variables	Estimate	RR	95% CI	*p* Value
LBW (<2.5 kg)	−0.573	**0.564**	**0.418–0.742**	**<0.001**
Prematurity	−0.167	**0.846**	**0.759–0.945**	**0.003**
Ingredients of complementary food				
Grains	−0.288	**0.750**	**0.639–0.877**	**<0.001**
Meets	0.245	**1.278**	**1.091–1.493**	**0.002**
Prolonged breastfeeding ^a^	0.139	**1.149**	**1.029–1.283**	**0.014**
Sugar-sweetened beverages ^b^, 3rd	0.179	1.196	0.988–1.437	0.061
Sugar-sweetened beverages ^b^, 4th	0.321	**1.378**	**1.143–1.652**	**<0.001**
Meals (≥4/day), 4th	0.312	1.367	0.936–1.937	0.091

^a^ ‘Prolonged breastfeeding’ refers to breastfeeding for 9 months or more. ^b^ ‘Sugar-sweetened beverages’ was defined as consuming more than 200 mL a day. Abbreviations: LBW, low birth weight; 3rd, questionnaire of National Health Screening Program for Infants and Children (NHSPIC) was carried out at 18–24 months; 4th, questionnaire of NHSPIC was carried out at 30–36 months. Bold style indicates when the *p* value < 0.05. This means it is statistically significant.

**Table 3:** Analysis by multivariable logistic regression on risk factors of eating behaviors, normal + overweight vs. obesity (Age of 4 years).

Variables	Estimate	RR	95% CI	*p* Value
LBW (<2.5 kg)	−0.570	**0.565**	**0.379–0.818**	**0.004**
Prematurity	0.268	1.308	0.922–1.814	0.119
Complementary food (≥ 3/day)	−0.144	**0.866**	**0.755–0.994**	**0.040**
Ingredients of complementary food				
Grains	−0.198	**0.820**	**0.675–0.993**	**0.045**
Meats	0.220	**1.246**	**1.025–1.508**	**0.026**
Other foods ^a^	0.112	1.119	0.967–1.298	0.134
Sugar-sweetened beverages ^b^, 3rd	0.185	1.203	0.952–1.503	0.113
Sugar-sweetened beverages ^b^, 5th	0.460	**1.583**	**1.262–1.967**	**<0.001**
Meals (≥4/day) ^c^, 5th	0.395	1.485	0.934–2.248	0.076

^a^ ‘other foods’ refers to sunsik, which is a cereal-based ready-to-drink Korean beverage, yogurt, or honey consumed during the first 9–12 months of life. ^b^ ‘Sugar-sweetened beverage’ was defined as consuming more than 200 mL a day. ^c^ ‘Meal (≥4/day)’ was defined as four or more meals a day. Abbreviations: LBW, low birth weight; 3rd, questionnaire of NHSPIC was carried out at 18–24 months; 5th, questionnaire of NHSPIC was carried out at 42–48 months. Bold style indicates a *p* value < 0.05. This means it is statistically significant.

**Table 4:** Analysis by multivariable logistic regression on risk factors of eating behaviors, normal + overweight vs. obesity (Age of 5 years).

Variables	Estimate	RR	95% CI	*p* Value
LBW (<2.5 kg)	−0.479	**0.619**	**0.415–0.886**	**0.013**
Complementary food (≥3/day) ^a^	−0.159	**0.853**	**0.743–0.980**	**0.025**
Ingredients of complementary food				
Meets	0.152	**1.164**	**1.011–1.340**	**0.034**
Sugar-sweetened beverages ^b^, 3rd	0.214	1.239	0.974–1.558	0.073
Sugar-sweetened beverages ^b^, 4th	0.234	1.263	0.990–1.594	0.054
Sugar-sweetened beverages ^b^, 5th	0.415	**1.514**	**1.192–1.903**	**<0.001**

^a^ ‘Complementary food (≥3/day) refers to intake complementary food at least three times a day during 9–12 months. ^b^ ‘Sugar-sweetened beverage’ was defined as consuming more than 200 mL a day. Abbreviations: LBW, low birth weight; 3rd, questionnaire of NHSPIC was conducted at 18–24 months; 4th, questionnaire of NHSPIC was conducted at 30–36 months; 5th, questionnaire of NHSPIC was carried out at 42–48 months. Bold style indicates a *p* value < 0.05. This means it is statistically significant.

## Data Availability

Data cannot be shared with others because it can only be verified by accessing an IP address that is restricted by security for a limited period of time under the permission of NHIS.

## Supplementary Materials

The following supporting information can be downloaded at: https://www.mdpi.com/article/10.3390/children11111297/s1. Table S1. Trends of normal-overweight-obesity at 3–5 years of age; Table S2. Analysis by multivariable logistic regression on risk factors of eating behaviors.
