# Supplementary material for: Trajectory of Obesity and the Impact of Eating Behaviors on Obesity in Preschool Children: A Nationwide Population-Based Cohort Study in Korea
Source: Children (Basel). 2024 Oct 27;11(11):1297. doi: 10.3390/children11111297 (PMC11592795; doi:10.3390/children11111297)
Supplement: Supplementary file 1 [file children-11-01297-s001.zip › children-3254888-supplementary.pdf]

**Supplementary Table S1**. Trends of normal-overweight-obesity at 3-5 years of age.

| Age 3 (n) | Age 4 (n) | Age 5 (n) |
|---|---|---|
| Normal<br>(n=13,184) | Normal<br>(n=12,301) | Normal (n=11,869)<br>Overweight (n=319)<br>Obesity (n=113) |
| | Overweight<br>(n=670) | Normal (n=461)<br>Overweight (n=137)<br>Obesity (n=72) |
| | Obesity<br>(n=213) | Normal (n=85)<br>Overweight (n=57)<br>Obesity (n=71) |
| Overweight<br>(n=1,984) | Normal<br>(n=1,292) | Normal (n=1107)<br>Overweight (n=136)<br>Obesity (n=49) |
| | Overweight<br>(n=491) | Normal (n=284)<br>Overweight (n=136)<br>Obesity (n=71) |
| | Obesity<br>(n=201) | Normal (n=49)<br>Overweight (n=61)<br>Obesity (n=91) |
| Obesity<br>(n=1,689) | Normal<br>(n=586) | Normal (n=473)<br>Overweight (n=83)<br>Obesity (n=30) |
| | Overweight<br>(n=477) | Normal (n=227)<br>Overweight (n=173)<br>Obesity (n=77) |
| | Obesity<br>(n=635) | Normal (n=77)<br>Overweight (n=160)<br>Obesity (n=398) |

**Supplementary Table S2-1.** Analysis by multivariable logistic regression on risk factors of eating behaviors, Normal vs Overweight + Obesity (Age of 3 years).

| Variables | Estimate | RR | 95% CI | *P* value |
|---|---|---|---|---|
| LBW (<2.5kg) | -0.414 | **0.661** | **0.545-0.796** | **<0.001** |
| Late complementary food[a] | 0.121 | **1.128** | **1.023-1.244** | **0.015** |
| Complementary food (≥ 3/day)[b] | -0.124 | **0.883** | **0.816-0.956** | **0.002** |
| Ingredients of complementary food | | | | |
| Fruits | -0.068 | **0.935** | **0.875-0.998** | **0.044** |
| Prolonged breastfeeding[c] | 0.063 | 1.065 | 0.985-1.151 | 0.116 |
| Sweetened beverage[d], 4$^{th}$ | 0.196 | **1.217** | **1.055-1.400** | **0.006** |
| Meals (≥ 4/day)[e], 4$^{th}$ | 0.319 | **1.375** | **1.038-1.804** | **0.024** |

a 'Late complementary food' refers to cases where complementary food was introduced after 7 months of age.
b 'Complementary food (≥ 3/day) refers to intake complementary food at least three times a day during 9-12 months.
c 'Prolonged breastfeeding' meant as breastfeeding for 9 months or more.
d 'Sweetened beverage' was defined as consuming more than 200 ml a day.
e 'Meal ((≥ 4/day)' was defined as four or more meals a day.
Abbreviations: LBW, low birth weight; 4$^{th}$, Questionnaire of NHSPIC was done at 30-36 months.
Bold style indicate when the *P* value < 0.05. This means it is statistically significant.

**Supplementary Table S2-2.** Analysis by multivariable logistic regression on risk factors of eating behaviors (Age of 4 years).

| **Variables** | **Estimate** | **RR** | **95% CI** | ***P* value** |
|---|---|---|---|---|
| LBW (<2.5kg) | -0.365 | **0.694** | **0.546-0.874** | **0.002** |
| Prematurity | 0.188 | 1.207 | 0.958-1.509 | 0.104 |
| Late complementary food[a] | 0.090 | 1.094 | 0.978-1.221 | 0.112 |
| Ingredients of complementary food | | | | |
| Grains | -0.151 | **0.860** | **0.758-0.974** | **0.018** |
| Meets | 0.134 | **1.143** | **1.003-1.302** | **0.045** |
| Unrecommended food[b] | 0.133 | **1.142** | **1.038-1.257** | **0.007** |
| Sweetened beverage[c], 3rd | 0.198 | **1.218** | **1.041-1.420** | **0.012** |
| Sweetened beverage[c], 5th | 0.294 | **1.342** | **1.141-1.571** | **<0.001** |

a 'Late complementary food' refers to cases where complementary food was introduced after 7 months of age.
b 'Unrecommended food' refers to sunsik, which is a cereal-based ready-to-drink Korean beverage, yogurt or honey consumed during the first 9-12 months of life.
c 'Sweetened beverage' was defined as consuming more than 200 ml a day.
Abbreviations: LBW, low birth weight; 3rd, Questionnaire of NHSPIC was done at 18-24 months; 5th, Questionnaire of NHSPIC was done at 42-48 months.
Bold style indicate when the *P* value < 0.05. This means it is statistically significant.

**Supplementary Table S2-3.** Analysis by multivariable logistic regression on risk factors of eating behaviors (Age of 5 years).

| Variables | Estimate | RR | 95% CI | *P* value |
|---|---|---|---|---|
| LBW (<2.5kg) | -0.477 | **0.621** | **0.466-0.815** | **<0.001** |
| Prematurity | 0.237 | 1.267 | 0.927-1.711 | 0.130 |
| Complementary food (≥ 3/day)[a] | -0.120 | **0.886** | **0.805-0.976** | **0.014** |
| Ingredients of complementary food | | | | |
| Meets | 0.106 | **1.111** | **1.007-1.226** | **0.035** |
| Sweetened beverage[b], 3rd | 0.130 | 1.139 | 0.958-1.346 | 0.134 |
| Sweetened beverage[b], 4th | 0.225 | **1.252** | **1.054-1.480** | **0.010** |
| Sweetened beverage[b], 5th | 0.314 | **1.368** | **1.150-1.621** | **<0.001** |
| Meals (≥ 4/day)[c], 4th | 0.492 | **1.635** | **1.191-2.207** | **0.002** |

a 'Complementary food (≥ 3/day) refers to intake complementary food at least three times a day during 9-12 months.
b 'Sweetened beverage' was defined as consuming more than 200 ml a day.
c 'Meal ((≥ 4/day)' was defined as four or more meals a day.
Abbreviations: LBW, low birth weight; 3rd, Questionnaire of NHSPIC was done at 18-24 months; 4th, Questionnaire of NHSPIC was done at 30-36 months; 5th, Questionnaire of NHSPIC was done at 42-48 months.
Bold style indicates when the *P* value < 0.05. This means it is statistically significant.
